# The natural compound sulforaphene, as a novel anticancer reagent, targeting PI3K-AKT signaling pathway in lung cancer

**DOI:** 10.18632/oncotarget.12307

**Published:** 2016-09-28

**Authors:** Ming Yang, Haiyong Wang, Mo Zhou, Weilin Liu, Pengqun Kuang, Hao Liang, Qipeng Yuan

**Affiliations:** ^1^ State Key Laboratory of Chemical Resource Engineering, Beijing Laboratory of Biomedical Materials, College of Life Science and Technology, Beijing University of Chemical Technology, Beijing, China

**Keywords:** sulforaphene, lung cancer, isothiocyanate, PI3K-AKT

## Abstract

Lung cancer is one of the leading causes of cancer death worldwide. Isothiocyanates from cruciferous vegetables been shown to possess anticarcinogenic activities in lung malignances. We previously found sulforaphene (4-methylsufinyl-3-butenyl isothiocyanate, SFE), one new kind of isothiocyanates, existing in a relative high abundance in radish seeds. An efficient methodology based on macroporous resin and preparative high-performance liquid chromatography was developed to isolate SFE in reasonably large quantities, high purity and low cost. However, it is still largely unclear whether SFE could function as an antineoplastic compound, especially in lung cancer. In this study, we systematically investigated the anti-cancer effects of SFE *in vitro* as well as its possible underling molecular mechanisms in lung cancer. The acute toxicity tests and pharmacokinetics tests for SFE were performed to evaluate its drugability in mice. Also, we evaluated the *in vivo* anti-cancer effects of SFE using nude Balb/C mice with lung cancer xenograft. SFE can induce apoptosis of multiple lung cancer celllines and, thus, inhibited cancer cell proliferation. Lung cancer cells treated with SFE exhibit significant inhibition of the PI3K-AKT signaling pathway, including depressed PTEN expression and inhibition of AKT phosphoralation. At well-tolerated doses, administration of SFE to mice bearing lung cancer xenografts leads to significant inhibitions of tumor growth. In summary, our work identifies SFE as a novel natural broad-spectrum small molecule inhibitor for lung cancer.

## INTRODUCTION

Lung cancer is one of the leading causes of cancer death worldwide, with over one million deaths each year [1]. There are about 80% of all lung cancer patients classified as non-small cell lung cancer (NSCLC) and the remaining 20% cases belong to small-cell lung cancer (SCLC). When diagnosed, about one third of NSCLC patients suffered from a locally advanced stage disease [2]. Treatment of these patients is usually based on a multidisciplinary strategy, including a combination of chemotherapy and radiotherapy. However, therapeutic efficacies were far from satisfactory with a 3-year overall survival being 10% to 20% [3]. Therefore, identification of new drugs for more selectively killing malignant cells while having little or no toxicity on their normal counterparts is warranted.

Accumulated epidemiological evidences suggest that diet rich in fruit and vegetables, especially cruciferous vegetables such as broccoli, cabbage, kale, cauliflower, and radish, is associated with a decreased risk of developing lung cancer [4–6]. This chemoprotective effect is closely related to isothiocyanates (ITCs), a group of hydrolysis products of glucosinolates, which are relatively unique in cruciferous vegetables [7, 8]. As one of the most studied ITCs, sulforaphane (4-methylsulfinybutyl isothiocyanate, SFA) was initially identified as the principal inducer of phase II enzymes [9] and has subsequently been shown to possess anticarcinogenic activities in many malignances [10–15]. Several randomized, placebo-controlled, double-blind phase I clinical trials or chemoprevention trials utilizing different sources of SFA demonstrated that SFA might be a promising anticancer agent with no significant toxicities [16–23]. However, the high cost for isolation of natural SFA from cruciferous vegetables limits its application. Interestingly, we found that sulforaphene (4-methylsufinyl-3-butenyl isothiocyanate, SFE), another kind of ITCs, exists in a relative high abundance in radish seeds and can be isolated in reasonably large quantities, high purity and low cost [24, 25]. It has been shown that SFE possesses pharmacological antimutagenicity, antimicrobial and antiviral activities [24, 25]. However, it is still not clear whether SFE could also function as an antineoplastic compound, especially in lung cancer. Therefore, in the current study, we investigated this anti-cancer effect of SFE *in vitro* and *in vivo* as well as its possible molecular mechanism.

## RESULTS

### Anti-cancer effects of SFE *in vitro*

**Figure 1:**
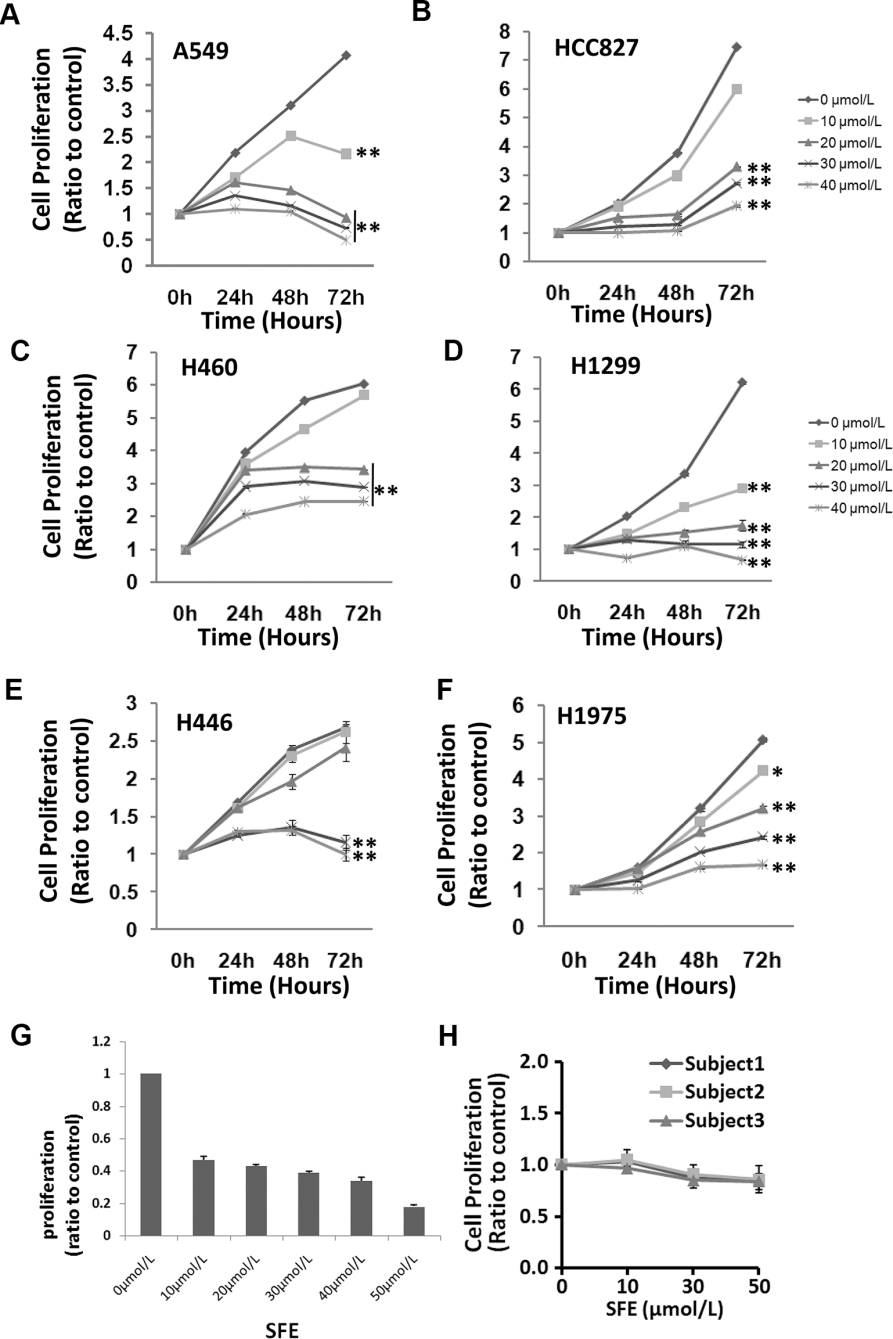
*In vitro* evaluation of anti-cancer effects of Sulforaphene (SFE) in multiple lung cancer cells as well as toxicity of SFE to human Peripheral blood mononucleated cells (PBMC) (**A**) Structure of SFE. (**B**–**G**) Anti-cancer effects of SFE in A549, H460, H1299, H446, HCC827 and H1975 lung cancer celllines. (H) Toxicity of SFE to human PBMC. All results of the mean of triplicate assays with standard deviation are presented. *P < 0.05, **P < 0.01.

Impact of SFE and SFA on cancer cell proliferation was examined in six human lung cancer cell lines (A549, H460, H446, HCC827, H1975 and H1299) and primary lung cancer cells isolated from surgery removed lung cancer tissues. It has been showed that SFE was effective in all lung cancer cell lines and primary lung cancer cells (Figure 1). The anti-cancer effects were more evident when cells treated with increased concentrations of SFE (range: 10 ∼ 40 μmol/L). As shown in Figure 1H, SFE seem to have little effects on proliferation of PBMCs from three different individuals. All these data demonstrate that SFE might be a natural compound with relatively pronounced anti-cancer activity in multiple kinds of lung cancer cells, but little toxicity to normal human cells.

### Induction of apoptosis by SFE

**Figure 2:**
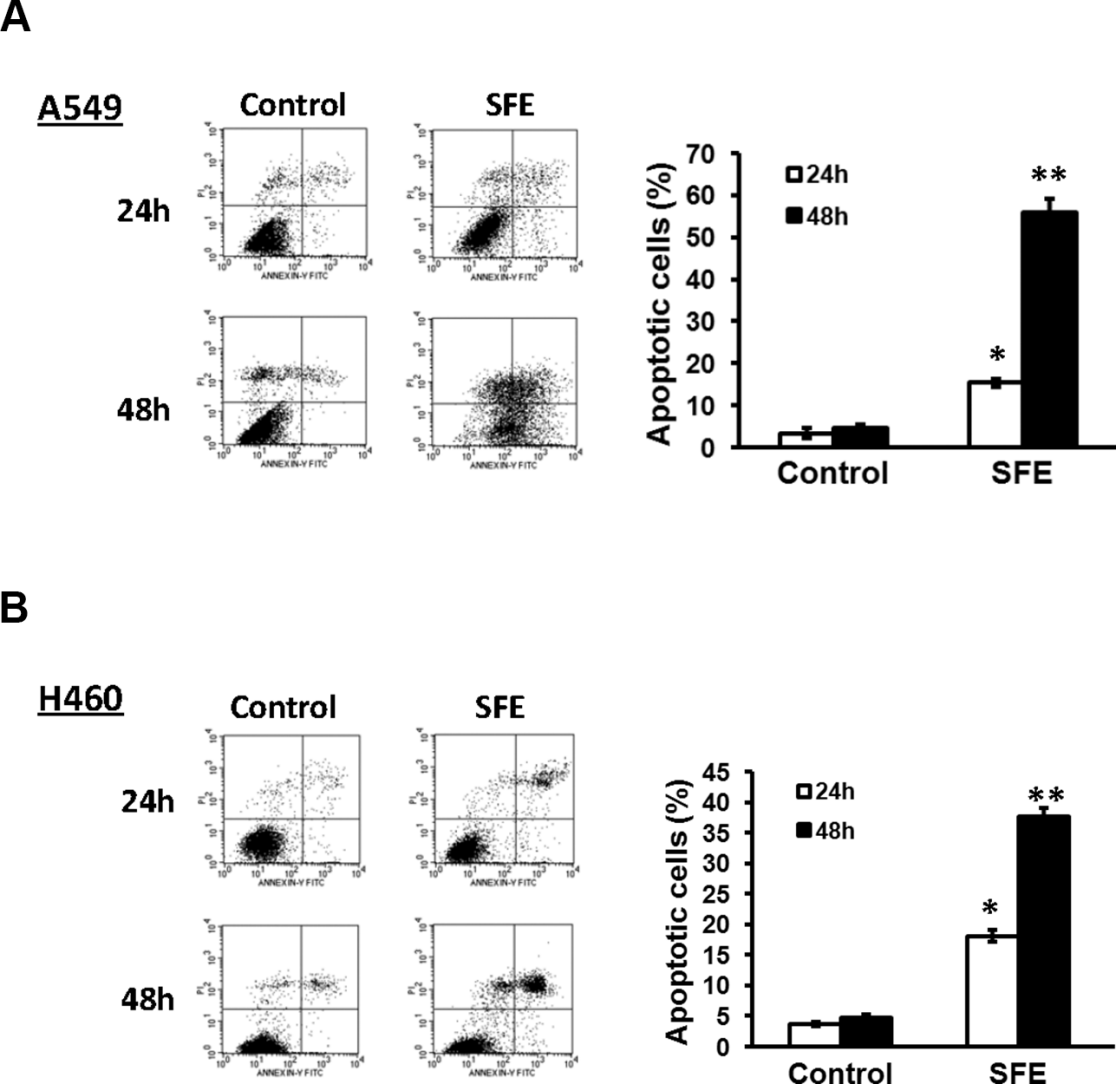
SFE inhibits lung cancer cell proliferation via inducing apoptosis (**A**) A549 cell apoptosis was examined at 24 h and 48 h after 30 μmol/L SFE treatment. (**B**) H460 cell apoptosis was examined at 24 h and 48 h after 30 μmol/L SFE treatment. All results of the mean of triplicate assays with standard deviation of the mean are presented. *P < 0.05, **P < 0.01.

To corroborate the hypothesis that the decreased cell number was due to the induction of cell death, we further determined impacts of SFE on apoptosis, using sterile H_2_O as the negtive control. After A549 and H460 cells were treated with 30 μmol/L SFE or sterile H_2_O for 24 h or 48 h, apoptosis was quantified with FCM (Figure 2A). There were 15.41 ± 0.99% or 55.92 ± 3.10% apoptotic A549 cells in the SFE treatment group at 24 h or 48 h. The apoptotic rates were 3.32 ± 1.20% and 4.70 ± 0.72% for cells incubated with sterile H_2_O at 24 h or 48 h. There were significantly more cells underwent apoptosis at 24 h or 48 h after SFE treatment compared to cells treated with sterile H_2_O (both *P* < 0.05). Similar results were observed in H460 cells. A total of 18.12 ± 0.98% or 37.70 ± 1.40% apoptotic H460 cells were detected at 24 h or 48 h after SFE treatment. However, there were only 3.66 ± 0.30% or 4.77 ± 0.54% apoptotic H460 cells at 24 h or 48 h after sterile H_2_O treatment. Significantly higher level of apoptosis was observed in the SFE group compared to the sterile H_2_O group (both *P* < 0.05). Our data suggested that SFE might be a more efficient compound inducing apoptosis of lung cancer cells. In sum, inhibition of proliferation by SFE might be largely owing to cell apoptosis.

### Inhibition of the PI3K-AKT cell signaling pathway by SFE

**Figure 3:**
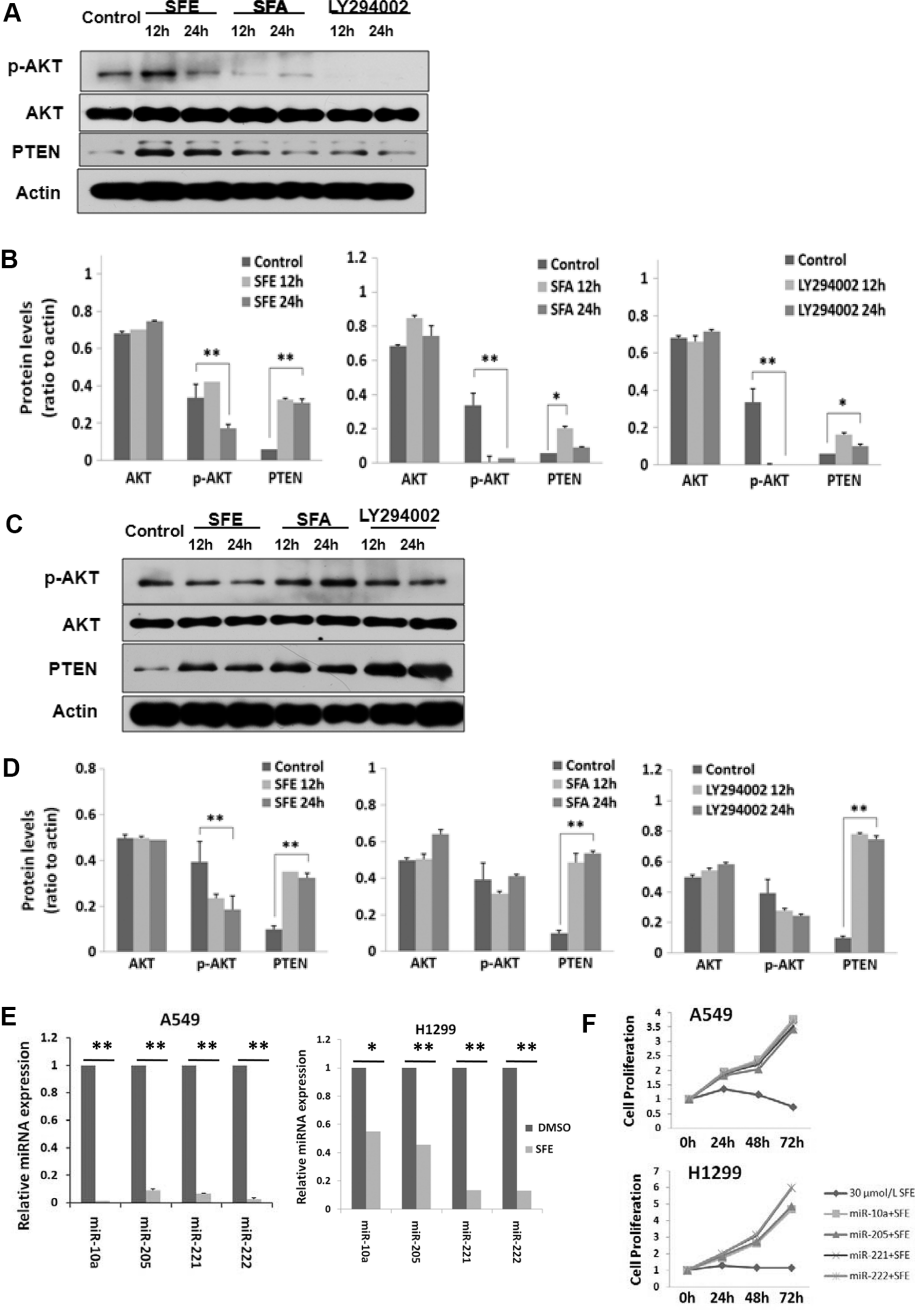
SFE inhibits the PI3K-AKT cell signaling pathway (**A**, **B**) SFE can significantly depress phosphorylated AKT levels and induce PTEN expression in A549 cells. (**C**, **D**) SFE can significantly depress phosphorylated AKT levels and induce PTEN expression in H460 cells. (E) SFE can significantly down-regulate miR-10a, miR-205, miR-221, and miR-222 expression in A549 cells. All results of the mean of triplicate assays with standard deviation of the mean are presented. *P < 0.05, **P < 0.01.

Accumulated evidences indicate that the PI3K-AKT signaling pathway plays an essential role in lung cancer development. Therefore, we examined whether SFE and SFA would impact the activation of this pathway with SFA and LY294002 (a known inhibitor of AKT phosphorylation) as controls. Although AKT protein expression levels did not change in both A549 and H460 cells with SFE or SFA treatment, phosphorylated AKT levels altered after exposure of these two ITCs (Figure 3). At 24 h after SFE treatment, there was about 50% decreased phosphorylation of AKT in both cell lines. Similar results could be found in cells treated with LY294002. Additionally, a 5.23-fold or 3.26-fold increased PTEN protein expression was observed in either A549 or H460 cells at 24 h after SFE treatment. All these results demonstrate that SFE might up-regulate tumor suppressor PTEN and, then, reducing AKT activation to inhibit PI3K-AKT signaling pathway in lung cancer. For SFA, although elevated PTEN expression and depressed AKT phosphorylation were found in A549 cells, similar results could not be repeated in H460 cells. The possible underline explanation is that SFA may inhibit different signaling pathway in various subtype of lung cancer cells. To further explore how PTEN was up-regulated in lung cancer cells by SFE, we detected multiple known PTEN-targeting miRNAs including miR-10a, miR-205, miR-221, and miR-222. Interestingly, we observed that all these miRNAs were significantly down-regulated After lung cancer A549 and H1299 cells treated with SFE. As a result, we speculate that a least part of the mechanism on up-regulation of PTEN after SFE treatment is due to suppressed expression of these miRNAs that targeting and inhibit PTEN expression (Figure 3E). Moreover, to validate the biological function of these miRNAs in lung cancer cells, we transfected A549 and H1299 cells with mimics of miR-10a, miR-205, miR-221, or miR-222 with or without treatment of SFE. In the rescue assays, we observed that the anti-cancer effects of SFE were significantly inhibited by miR-10a, miR-205, miR-221, or miR-222 (Figure 3F).

### Acute toxicity testing in mice

**Figure 4:**
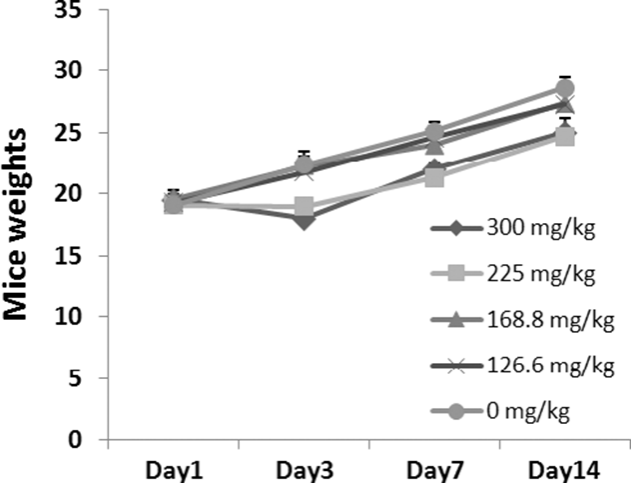
Acute toxicity testing of SFE in mice Forty-eight mice were treated with SFE at 5 different doses of vehicle control or 400, 300, 225, 168.8 and 126.6 mg/kg via lavage administration (*n* = 8 for each group). Weight changes of mice were showed.

After 14 days, all eight mice treated with 126.6 mg/kg SFE survived the treatment period. However, there were 8, 7, 4 or 2 animals treated with 400, 300, 225 or 168.8 mg/kg SFE died in 24 h after administration. Moreover, one mouse treated with 225 or 168.8 mg/kg SFE died in 48 h. For mice in the 126.6 mg/kg SFE group, no physical or abnormal changes was observed in sleep patterns, behavior patterns, fur, skin, eyes, mucus membranes, tremors or salivation. The LD_50_ value of SFE was 202.7 ± 31.5 mg/kg. There were no differences between mice treated with 126.6 mg/kg SFE or sterile H_2_O as shown in Figure 4 (*P* > 0.05).

### Pharmacokinetics in mice

**Figure 5:**
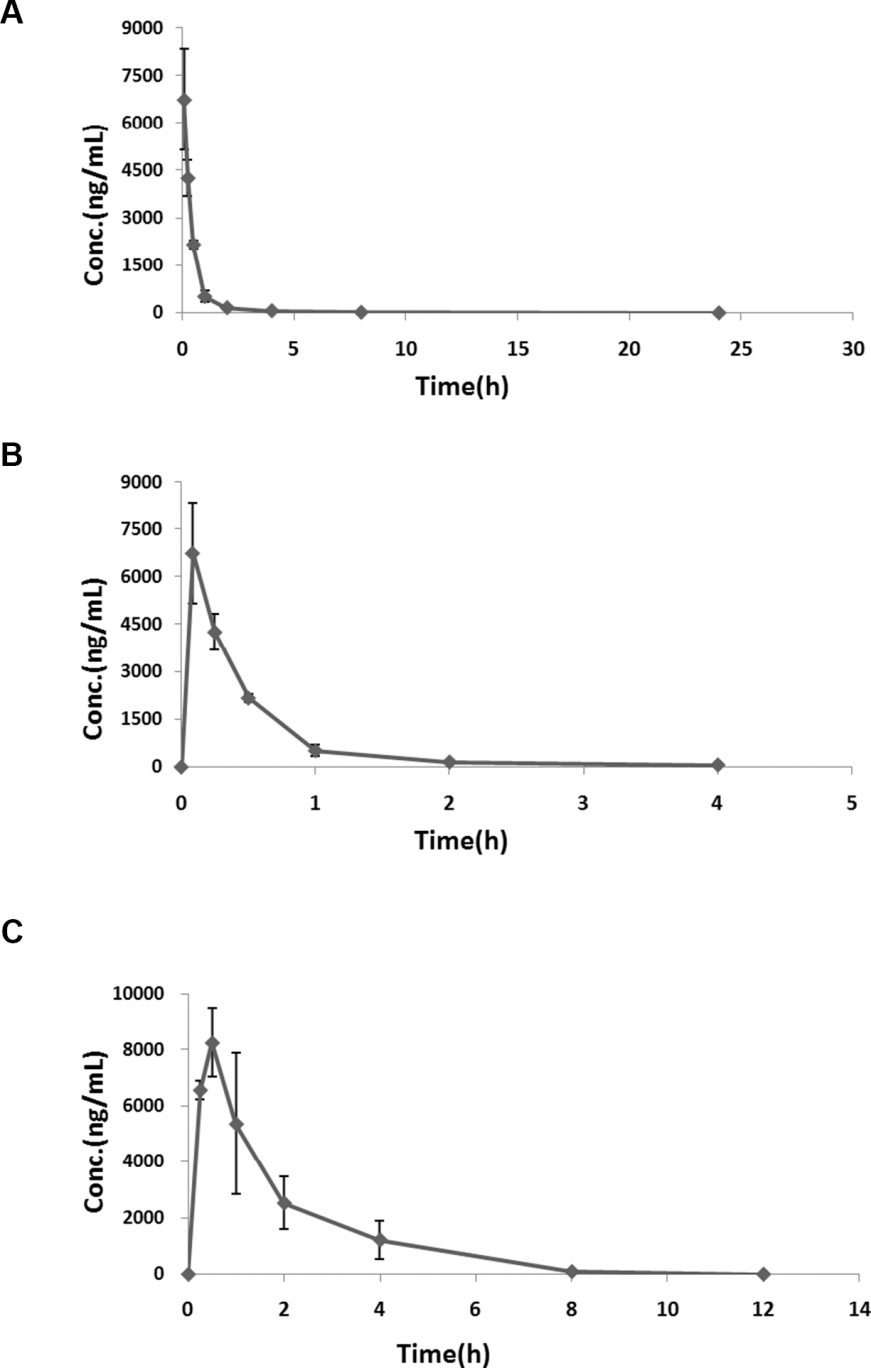
*In vivo* pharmacokinetics assays (**A**, **B**) The pharmacokinetic properties of SFE were evaluated in ICR mice after intravenous injection. (**C**) The pharmacokinetic properties of SFE were evaluated in ICR mice after oral gavage. Three mice were measured at each data point and all results of the mean with standard deviation are presented.

The pharmacokinetic properties of SFE were evaluated in ICR mice after intravenous injection or oral gavage. Pharmacokinetics analysis was performed by treating mice with single doses of 10 mg/kg SFE via intravenous injection or a single dose of 50 mg/kg SFE via oral gavage. Plasma was obtained at specified time points, and concentrations of SFE were determined by LC-MS/MS. As shown in Figure 5, SFE was absorbed rapidly and reached maximum plasma concentrations (C_max_) of 6.75 ± 1.61 mg/mL after intravenous injection or 8.25 ± 1.20 mg/mL at 30 min after oral administration. Elimination half-lives (T_1/2_) were 1.05 ± 0.44 h for intravenous injection or 1.14 ± 0.26 h for oral administration. The SFE elimination rate constant (K_el_) in mice was 0.75 ± 0.32/h via intravenous injection or 0.63 ± 0.16/h via oral administration, respectively.

### *In vivo* anti-cancer effects of SFE

**Figure 6:**
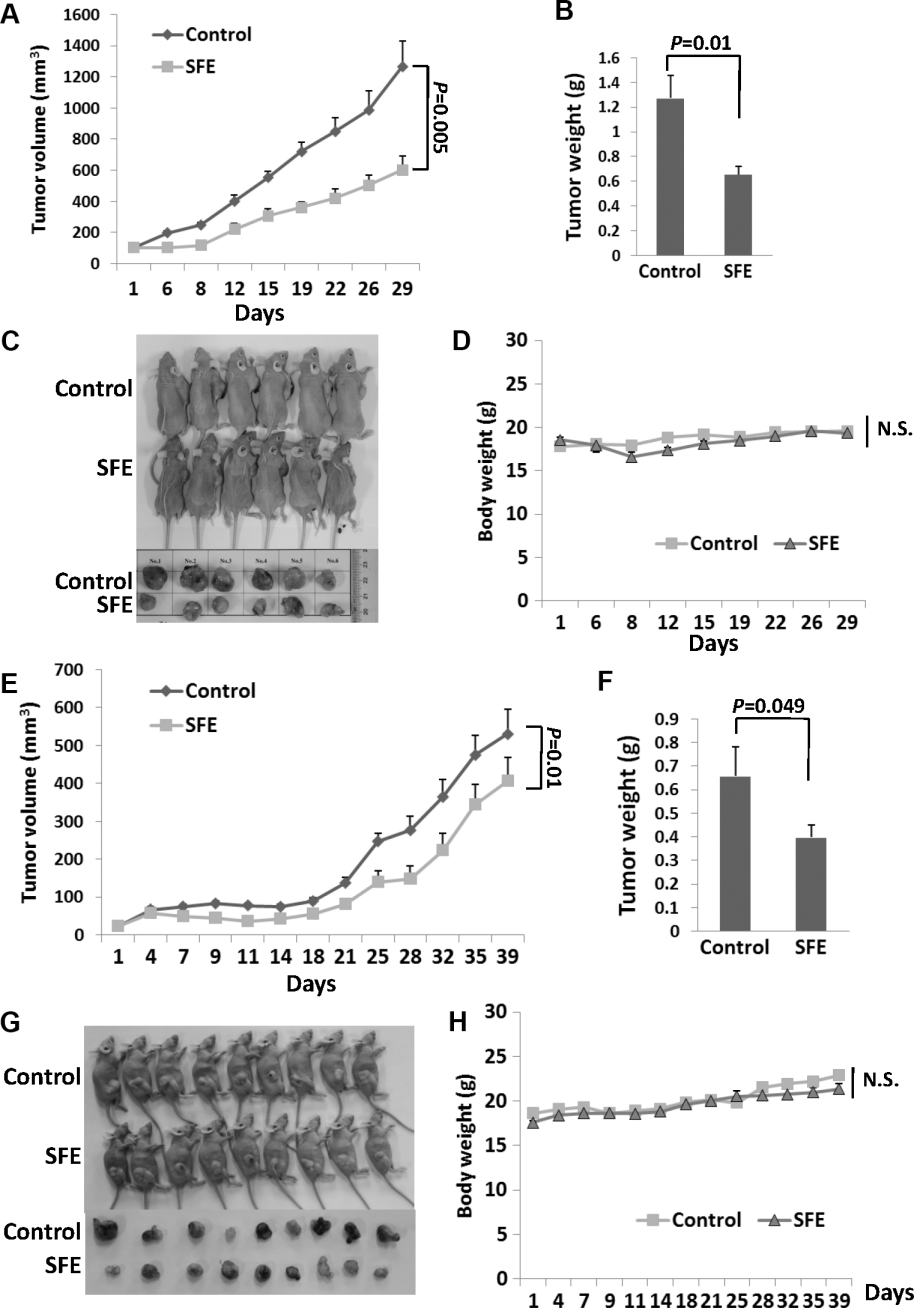
*In vivo* characterization of the anticancer potential SFE on human xenograft lung cancer tumors (**A**–**D**) Nude Balb/C mice with A549 xenograft treated with SFE via injection administration. (**E**–**H**) Nude Balb/C mice with A549 xenograft treated with SFE via oral administration. (A**–**C, E–G) The growth of tumors from SFE treatment was inhibited significantly compared with that of tumors treated with vehicle control. (D, H) There were no significant differences of mice weight between SFE or vehicle control treated groups. N.S., not significant.

To evaluate the anti-cancer potential of the SFE *in vivo*, we used nude Balb/C mice with A549 xenograft treated with SFE via injection or oral administration (Figure 6). Nude mice received SFE treatment via injection in a dose of 50 mg/kg or via oral gavage in a dose of 100 mg/kg. There were statistically significant decreases in tumor growth of the SFE treated group via either injection or oral gavage compared with the control group. At day 29 posttreatment via injection, the average tumor volume of the SFE group was 47.6% of that of the solvent control group (*P* = 0.005). Similarly, the average tumor weight of mice treated with SFE was 51.4% of that of the solvent control group (*P* = 0.01). However, no significant mice body weight changes were observed between two groups (*P* > 0.05). Similar decreases of the tumor volume and tumor weight in mice orally treated with SFE were found compared with mice treated orally with solvent. However, significant higher inhibition efficiency in mice treated with SFE via injection was observed compared to the oral treatment (inhibition rate of tumor weight: 51.4% vs. 60.5%). These results illustrate that the intake of SFE does have inhibitory effects on lung cancer tumor growth *in vivo*.

## DISCUSSION

In the current study, we investigated the anti-cancer activity of SFE against multiple lung cancer cell lines *in vitro* and *in vivo* with a xenograft mouse model. It has been showed that SFE exhibited antiproliferative effects against multiple kinds of lung cancer cells *ex vivo* and *in vivo* while having little adverse impacts. The underline mechanism of SFE's anti-cancer activities might be its up-regulate tumor suppressor PTEN and, then, reducing AKT activation to inhibit PI3K-AKT signaling pathway. These findings are consistent to the role of SFE in different cancers which have been reported recently [27–29].

Although sharing some similar environmental etiological factor such as heavy tobacco smoking, NSCLC and SCLC do show different biological behaviors and have diverse treatment strategies in clinic. We evaluated the anti-cancer effects of SFE using both kinds of lung cancer cells (A549, NSCLC; H460, large cell lung cancer; H446, SCLC; HCC827, adenocarcinoma/NSCLC; H1975, adenocarcinoma/NSCLC and H1299, NSCLC/*TP53* null) and found similar activities of SFE for these cells. These results indicate that SFE might be a common antineoplastic compound for lung cancer.

As a critical tumor suppressor and phosphatase, PTEN is implicated in a wide variety of human malignancies [26, 30]. Through dephosphorylating its main substrates (inositol phospholipids), PTEN could depress the PI3K-AKT signaling pathway [26, 30]. In this pathway, AKT is a key regulator of cell survival and apoptosis [26, 30]. AKT is activated by phospholipid binding and activation loop phosphorylation at Thr308 by PDK1 [26, 30]. AKT promotes survival of malignant cells by inhibiting apoptosis by phosphorylating and inactivating several targets, including Bad, forkhead transcription factors, c-Raf and caspase-9. Consistent to the roles of PTEN and AKT, our findings that SFE could inhibit lung cancer growth and induce apoptosis of cancer cells through down-regulation of PTEN expression and inhibition of AKT activation.

In summary, SFE is a potential small natural compound for the treatment of lung cancer with a mechanism of potent inhibition of the PI3K-AKT signaling pathway. At well-tolerated doses, SFE has significant anti-cancer activities *in vivo* to the lung cancer xenograft model as a single agent.

## MATERIALS AND METHODS

### Preparation of SFE and SFA

SFE were isolated from radish seeds (yi tian bai yu xue), which were purchased from Beijing Tongrentang Co., LTD (Beijing, China). Broccoli seeds (kindly provided by Vegetables and Flowers Institute, China Academy of Agriculture Science), were used for SFA preparation. SFE (> 98%) was separated and purified by high-speed counter current chromatography (HSCCC) in our laboratory as reported previously [24, 25]. The procedure of SFA (> 98%) preparation included solvent extraction of autolyzed seed meal, followed by separation by solid phase extraction and purification by preparative high-performance liquid chromatography (HPLC) [31]. The purity and chemical structure of SFE and SFA were identified by analytical HPLC, ESI-MS and NMR [24, 25, 31]. SFE and SFA were dissolved in vehicle (sterile water) for cell culture and animal studies.

### Cell culture

Human lung cancer cells (A549, H460, H446, HCC827, H1975 and H1299) were obtained from from the Cell Resource Center, Peking Union Medical College (which is the headquarter of National Infrastructure of Cell Line Resource, NSTI) on May 20, 2015. The cell line was checked free of mycoplasma contamination by PCR and culture. Its species origin was confirmed with PCR. The identity of the cell line was authenticated with STR profiling (FBI, CODIS). All the results can be viewed on the website (http://cellresource.cn). Cells were cultured in RPMI 1640 containing 10% fetal bovine serum (FBS) in the presence of 100 units/mL penicillin and 0.1 g/L streptomycin. Cells were incubated at 37°C with 95% air and 5% carbon dioxide. All cells were used in experiments during the linear phase of growth.

### Peripheral blood mononucleated cell (PBMC) isolation and culture

Blood samples were collected from 3 healthy volunteers (2 males and 1 female) with no history of malignant disease. A total of 5 × 10^6^ PBMC/mL were isolated by Ficoll (Sigma-Aldrich) density-gradient centrifugation and suspended in RPMI 1640 medium. PBMCs were then cultured in RPMI 1640 containing 10% FBS as described above. Informed consent was obtained from each subject at recruitment.

### Cell proliferation assays

Lung cancer cells in 96-well plates were treated with 100 μL culture media plus vehicle control or SFE and incubated for 24, 48 and 72 hours (h). SFE was tested at 10, 20, 30 and 40 μmol/L concentrations. PBMCs were only incubated with 100 μL culture media plus water, SFE (10, 30 and 50 μmol/L) and incubated for 24 h due to their limited proliferation ability. Cell viability was examined using the MTT [3-(4,5-Dimethylthiazol-2-yl)-2,5-diphenyl-2H-tetrazolium Bromide] Cell Viability Assay according to the manufacturer's instructions (Catalog no. 0793; Amresco) at the end of the drug exposure duration. There were control wells containing culture medium without cells in all plates to obtain a value for background luminescence, which was utilized for normalization of the test sample readings. Data were presented as mean ± SD for five replications.

### Annexin V/propidium iodide apoptosis assay and determination of DNA content

A549 or H460 cells were cultured in 6-well plates at a concentration of 150,000 per well and allowed to adhere for 24 hours. Media plus vehicle control or 30 μmol/L SFE were used to treat lung cancer cells for 24 h or 48 h. Following treatments, nonadherent and adherent cells were collected and apoptosis was determined using the Alexa Fluor^®^ 488 annexin V/Dead Cell Apoptosis Kit (Catalog no. V13245; Invitrogen) by the FACSCalibur flow cytometer (FCM) (BD Biosciences), according to the manufacturer's instructions. For DNA content determination, A549 or H460 cells were harvested 48 h after treatment and washed twice with cooled PBS containing 10% FBS, then suspended with PBS containing 3% FBS and 70% ethanol. Cells were treated with 1 mg/mL RNaseA and stained with 5 mg/mL propidium iodide (Sigma). The analysis was performed by the FACSCalibur FCM (BD Biosciences) to determine the percentage of cells with 2N DNA, at S phase, and with 4 N DNA content.

### Real-time qRT-PCR

Total cellular RNAs were extracted from A549 cells. Stem-loop RT-PCR primers for human miR-10a, miR-205, miR-221, miR-222, and U6 were synthesized by Ribobio (Guangzhou, China). For miR-10a, miR-205, miR-221, miR-222, and U6 detection, RNA samples were reverse-transcribed into cDNA using Revert Ace transcriptase by specific stem-loop RT primers according to the manufacturer's instruction. Transcript levels were measured against an endogenous control by quantitative PCR using the SYBR Green I fluorogenic dye using the Mastercycler ep realplex system (Eppendorf, Hamburg, Germany).

### Western blotting

Total proteins from various lung cancer cells treated with vehicle control, SFE, SFA or LY294002 were extracted with radioimmunoprecipitation assay (RIPA) buffer [50 mmol/L Tris-HCl (pH 7.4), 150 mmol/L NaCl, 1% NP40, 0.5% sodium deoxycholate, 0.1% SDS, 5 mmol/L EDTA]. Twenty micrograms of each protein extract were resolved on 10% SDS-PAGE and transferred to Hybond-C nitrocellulose. The levels of AKT, phosphorylated AKT, and PTEN expression were determined by rabbit polyclonal anti-AKT antibody (Catalog no. 9272; Cell Signaling), rabbit polyclonal phosphorylated AKT (Ser473) antibody (Catalog no. 9271; Cell Signaling), and rabbit monoclonal anti-PTEN antibody (Catalog no. 5384; Cell Signaling), with Pierce ECL Western Blotting Substrate (Catalog no. 32209; Thermo). β-actin expression levels were measured using a monoclonal anti-β-actin (A 3853; Sigma) as a loading control.

### Acute toxicity testing in mice

The acute toxicity test for SFE was performed according to the up-and-down method [32]. After fasted overnight, forty-eight mice were treated with SFE at 5 different doses of vehicle control or 400, 300, 225, 168.8 and 126.6 mg/kg via lavage administration (*n* = 8 for each group). After administration, the animals were observed carefully for any gross effects or mortality. Weights, symptoms and deaths were recorded. Autopsies were done if animal died. After 14 days, all live mice were sacrificed for autopsy to observe whether all organs were normal.

### *In vivo* pharmacokinetics assays

Eighteen female ICR mice were used in the pharmacokinetics analyses. Among them, nine mice were treated with a single dose of 10 mg/kg SFE via intravenous injection in a volume of 5 mL/kg. The other nine were treated with a single dose of 50 mg/kg SFE via oral gavage in a volume of 10 mL/kg. In the intravenous injection group, about 50 μL saphenous vein blood was collected into lithium-heparin plasma separator tubes from 3 mice per time point over a 24 h period at 0, 5, 15, 30, 60, 120, 240, 480 and 1440 minutes post dose. In the oral gavage group, about 50 μL saphenous vein blood was collected into lithium-heparin plasma separator tubes from 3 mice per time point over a 24 h period at 0, 15, 30, 60, 120, 240, 480, 720 and 1440 minutes post dose. Briefly, the peak area ratios of the test compound and verapamil (internal standard) were determined by tandem quadrupole detector (AB Sciex API 4000). Separation was performed on an Agilent ZORBAX SB-C18 Column (50 × 2.1 mm, 3.5 μm) eluted at a flow rate of 0.35 mL/min. Mobile phase A was 0.1% (v/v) formic acid in water, and mobile phase B was 0.1% (v/v) formic acid in acetonitrile. The gradient consisted of 25% of mobile phase B for 0.5 min after injection and increased linearly to 90% B from 0.5 to 1.76 min, and from 90% B to 25% B from 1.76 to 3.2 min. SFE was detected by positive ion spray in the multiple-reaction monitoring mode using predetermined parent/product mass transition ion pairs. The pharmacokinetic parameters were calculated using Microsoft Excel with pharmacokinetic add-in functions from Usansky et al. (https://www.boomer.org/pkin/soft.html). Parameters were calculated using plasma concentration time data for composite averages.

### Xenograft tumor growth

Five-week old female nude BALB/c mice were purchased from Vital River Laboratory (Beijing, China). To evaluate the anti-cancer effects of SFE *in vivo*, 1 × 10^7^ A549 cells were inoculated subcutaneously into fossa axillaris of nude mice. After 20 days, thirty xenograft mice with similar tumor volume were elected (tumor volumes = 105 mm^3^, range: 82.0 ∼ 126 mm^3^). Thirty tumor-bearing mice were divided into four groups for SFE or solvent control administration. In details, eighteen mice were orally treated with 100 mg/kg SFE in a volume of 10 mL/kg (*n* = 9) or solvent control in the same volume (*n* = 9) twice a day. Twelve mice were treated with 50 mg/kg SFE (*n* = 6) or solvent control (*n* = 6) via intravenous injection twice a day. After one week, the intravenous injection mice were treated with the same dose of SFE or solvent control (*n* = 6) via intraperitoneal injection twice a day until the end of the study. Tumor volumes and mice weights were measured. Tumor volumes were calculated using the following formula: 0.5 × length (mm) × width (mm) × width (mm). All procedures involving mice were approved by the Institutional Review Board of Beijing University of Chemical Technology.

### Statistics

To assess statistical significance, values were compared with controls with either Student's *t* test or one-way ANOVA. A *P value* of less than 0.05 was used as the criterion of statistical significance, and all statistical tests were two-sided. All analyses were performed with SPSS software package (Version 16.0, SPSS Inc.).

## References

[1] Siegel R, Naishadham D, Jemal A (2013). Cancer statistics, 2013. CA Cancer J Clin.

[2] Gandara D, Narayan S, Lara PN, Goldberg Z, Davies A, Lau DH, Mack P, Gumerlock P, Vijayakumar S (2005). Integration of novel therapeutics into combined modality therapy of locally advanced non-small cell lung cancer. Clin Cancer Res.

[3] Eberhardt W, Pöttgen C, Stuschke M (2006). Chemoradiation paradigm for the treatment of lung cancer. Nat Clin Pract Oncol.

[4] Lam TK, Gallicchio L, Lindsley K, Shiels M, Hammond E, Tao XG, Chen L, Robinson KA, Caulfield LE, Herman JG, Guallar E, Alberg AJ (2009). Cruciferous vegetable consumption and lung cancer risk: a systematic review. Cancer Epidemiol Biomarkers Prev.

[5] Lam TK, Ruczinski I, Helzlsouer KJ, Shugart YY, Caulfield LE, Alberg AJ (2010). Cruciferous vegetable intake and lung cancer risk: a nested case-control study matched on cigarette smoking. Cancer Epidemiol Biomarkers Prev.

[6] Wu QJ, Xie L, Zheng W, Vogtmann E, Li HL, Yang G, Ji BT, Gao YT, Shu XO, Xiang YB (2013). Cruciferous vegetables consumption and the risk of female lung cancer: a prospective study and a meta-analysis. Ann Oncol.

[7] Abdull Razis AF, Noor NM (2013). Cruciferous vegetables: dietary phytochemicals for cancer prevention. Asian Pac J Cancer Prev.

[8] Hecht SS (1999). Chemoprevention of cancer by isothiocyanates, modifiers of carcinogen metabolism. J Nutr.

[9] Zhang Y, Talalay P, Cho CG, Posner GH (1992). A major inducer of anticarcinogenic protective enzymes from broccoli: isolation and elucidation of structure. Proc Natl Acad Sci USA.

[10] Zhang Y, Kensler TW, Cho CG, Posner GH, Talalay P (1994). Anticarcinogenic activities of sulforaphane and structurally related synthetic norbornyl isothiocyanates. Proc Natl Acad Sci USA.

[11] Gerhäuser C, You M, Liu J, Moriarty RM, Hawthorne M, Mehta RG, Moon RC, Pezzuto JM (1997). Cancer chemopreventive potential of sulforamate, a novel analogue of sulforaphane that induces phase 2 drug-metabolizing enzymes. Cancer Res.

[12] Kim DH, Sung B, Kang YJ, Hwang SY, Kim MJ, Yoon JH, Im E, Kim ND (2015). Sulforaphane inhibits hypoxia-induced HIF-1α and VEGF expression and migration of human colon cancer cells. Int J Oncol.

[13] Abbas A, Hall JA, Patterson WL, Ho E, Hsu A, Al-Mulla F, Georgel PT (2016). Sulforaphane modulates telomerase activity via epigenetic regulation in prostate cancer cell lines. Biochem Cell Biol.

[14] Lubecka-Pietruszewska K, Kaufman-Szymczyk A, Stefanska B, Cebula-Obrzut B, Smolewski P, Fabianowska-Majewska K (2015). Sulforaphane Alone and in Combination with Clofarabine Epigenetically Regulates the Expression of DNA Methylation-Silenced Tumour Suppressor Genes in Human Breast Cancer Cells. J Nutrigenet Nutrigenomics.

[15] Wang L, Tian Z, Yang Q, Li H, Guan H, Shi B, Hou P, Ji M (2015). Sulforaphane inhibits thyroid cancer cell growth and invasiveness through the reactive oxygen species-dependent pathway. Oncotarget.

[16] Myzak MC, Tong P, Dashwood WM, Dashwood RH, Ho E (2007). Sulforaphane retards the growth of human PC-3 xenografts and inhibits HDAC activity in human subjects. Exp Biol Med (Maywood).

[17] Shapiro TA, Fahey JW, Dinkova-Kostova AT, Holtzclaw WD, Stephenson KK, Wade KL, Ye L, Talalay P (2006). Safety, tolerance, and metabolism of broccoli sprout glucosinolates and isothiocyanates: a clinical phase I study. Nutr Cancer.

[18] Kensler TW, Chen JG, Egner PA, Fahey JW, Jacobson LP, Stephenson KK, Ye L, Coady JL, Wang JB, Wu Y, Sun Y, Zhang QN, Zhang BC (2005). Effects of glucosinolate-rich broccoli sprouts on urinary levels of aflatoxin-DNA adducts and phenanthrene tetraols in a randomized clinical trial in He Zuo township, Qidong, People's Republic of China. Cancer Epidemiol Biomarkers Prev.

[19] Liang H, Lai B, Yuan Q (2008). Sulforaphane induces cell-cycle arrest and apoptosis in cultured human lung adenocarcinoma LTEP-A2 cells and retards growth of LTEP-A2 xenografts *in vivo*. J Nat Prod.

[20] Conaway CC, Wang CX, Pittman B, Yang YM, Schwartz JE, Tian D, McIntee EJ, Hecht SS, Chung FL (2005). Phenethyl isothiocyanate and sulforaphane and their N-acetylcysteine conjugates inhibit malignant progression of lung adenomas induced by tobacco carcinogens in A/J mice. Cancer Res.

[21] Jin CY, Moon DO, Lee JD, Heo MS, Choi YH, Lee CM, Park YM, Kim GY (2007). Sulforaphane sensitizes tumor necrosis factor-related apoptosis-inducing ligand-mediated apoptosis through downregulation of ERK and Akt in lung adenocarcinoma A549 cells. Carcinogenesis.

[22] Mi L, Wang X, Govind S, Hood BL, Veenstra TD, Conrads TP, Saha DT, Goldman R, Chung FL (2007). The role of protein binding in induction of apoptosis by phenethyl isothiocyanate and sulforaphane in human non-small lung cancer cells. Cancer Res.

[23] Kalpana Deepa Priya D, Gayathri R, Sakthisekaran D (2011). Role of sulforaphane in the anti-initiating mechanism of lung carcinogenesis *in vivo* by modulating the metabolic activation and detoxification of benzo(a)pyrene. Biomed Pharmacother.

[24] Kuang P, Song D, Yuan Q, Lv X, Zhao D, Liang H (2013). Preparative separation and purification of sulforaphene from radish seeds by high-speed countercurrent chromatography. Food Chem.

[25] Kuang P, Song D, Yuan Q, Yi R, Lv X, Liang H (2013). Separation and purification of sulforaphene from radish seeds using macroporous resin and preparative high-performance liquid chromatography. Food Chem.

[26] Hopkins BD, Parsons RE (2014). Molecular pathways: intercellular PTEN and the potential of PTEN restoration therapy. Clin Cancer Res.

[27] Mondal A, Biswas R, Rhee YH, Kim J, Ahn JC (2016). Sulforaphene promotes Bax/Bcl2, MAPK-dependent human gastric cancer AGS cells apoptosis and inhibits migration via EGFR, p-ERK1/2 down-regulation. Gen Physiol Biophys.

[28] Kaczyńska A, Świerczyńska J, Herman-Antosiewicz A (2015). Sensitization of HER2 Positive Breast Cancer Cells to Lapatinib Using Plants-Derived Isothiocyanates. Nutr Cancer.

[29] Biswas R, Ahn JC, Kim JS (2015). Sulforaphene Synergistically Sensitizes Cisplatin via Enhanced Mitochondrial Dysfunction and PI3K/PTEN Modulation in Ovarian Cancer Cells. Anticancer Res.

[30] Song MS, Salmena L, Pandolfi PP (2012). The functions and regulation of the PTEN tumour suppressor. Nat Rev Mol Cell Biol.

[31] Liang H, Li C, Yuan Q, Vriesekoop F (2007). Separation and purification of sulforaphane from broccoli seeds by solid phase extraction and preparative high-performance liquid chromatography. J Agric Food Chem.

[32] Bruce RD (1985). An up-and-down procedure for acute toxicity testing. Fundam Appl Toxicol.

